# Investigating perceived stress and pain reduction following brief Reiki sessions in high-stress communities: an exploratory study

**DOI:** 10.3389/fpsyg.2025.1625414

**Published:** 2025-11-24

**Authors:** Heather McCutcheon, Sana Kausar Habiya

**Affiliations:** 1 Reiki Brigade NFP, Chicago, IL, United States; 2 Department of Health Sciences and Physical Education, Northeastern Illinois University (NEIU), Chicago, IL, United States

**Keywords:** Reiki, stress, pain, frontline professionals, mental health, biofield therapy, nonviolence

## Abstract

**Background:**

Reiki is a non-invasive modality that has shown significant promise in reducing stress and pain levels across diverse populations. Its use is expanding in hospitals where reported benefits include improvement in both physical and psychological parameters. Prior research suggests Reiki offers benefits as a complementary intervention for stress and pain, yet the impact of brief, community-based sessions has little evidence to date. This study focuses on brief, community-based Reiki sessions in reducing stress and pain levels in high-stress communities in Chicago.

**Objectives:**

To analyze changes in self-reported stress and pain levels before and after a single, ten-minute Reiki session among high-stress groups in community settings in Chicago.

**Methods:**

In this exploratory study, data from 59 events between September 2022–December 2024 was analyzed to evaluate the impact of ten-minute Reiki sessions on participants’ self-reported stress and pain levels. Quantitative measurements were determined using a 1–10 scale accompanied by facial expression visuals to facilitate understanding. For qualitative measures, participants were asked open-ended questions post-intervention such as, “How do you feel?” or “How did that go?” and feedback was recorded. All data were de-identified. No demographic or baseline health information was collected. Quantitative data were analyzed using descriptive statistics and significance testing. Qualitative responses were summarized descriptively and categorized into themes based on word occurrence frequency.

**Results:**

Reiki sessions were provided to 1,724 members of high-stress communities throughout Chicago. The majority of participants reported significant change in stress (72.62% reduction overall) and pain (63.34% reduction overall) following a single ten-minute session. Quantitative results were statistically significant, with a *p*-value of <0.01 in all categories. Most participants felt very relaxed and reported feeling less pain and profound surprise at the positive outcome post-session.

**Conclusion:**

This study has provided valuable insights into how participants perceive the impact of Reiki in reducing both stress and pain levels among individuals in high-stress communities who received a single, ten-minute Reiki session. Sessions took place in a variety of non-clinical settings. More rigorous research is needed to evaluate the impact of short Reiki sessions in non-clinical settings, and repeated sessions over time.

## Highlights

- This paper evaluates data gathered from ten-minute Reiki sessions administered to members of high-stress communities in a variety of settings.
- The results indicate Reiki reduced participants’ perceptions of stress and pain, and Reiki may be beneficial as a means for trained individuals outside the healthcare system to address stress and pain for members of their own communities.

## 1 Introduction

Certain segments of the population experience higher-than-average levels of stress due to their jobs, such as first responders (Bryant, 2022), or specific, challenging, life circumstances, such as students during exams (Fritz et al., 2021). Stress is strongly related to anxiety in terms of neural and behavioral bases (Daviu et al., 2019). According to a 2024 poll by the American Psychiatric Association, 43% of American adults said they felt more anxious than they did the previous year, up from 37% in 2023 and 32% in 2022. Respondents cited current events, including the economy, the U.S. election, and gun violence as reasons for the rise (Connors, 2024).

Commonly accepted means of stress management currently include cognitive behavioral therapy (Curtiss et al., 2021), pharmacology (Astill Wright et al., 2019), and mindfulness-based stress reduction (Hoge et al., 2023). But not everyone who would benefit from these interventions utilizes them. Barriers that prevent first responders from seeking out mental health care may include a lack of awareness, reluctance to show weakness, fear of breach of confidentiality (Jones et al., 2020), negative stigma, fear of negative career impact, and accessibility (Bryant, 2022; Haugen et al., 2017). Yet in an unsettling trend, seven Chicago police officers died by suicide in 2022, up from four in 2021, and two in 2020 (ABC7 Chicago Digital Team, 2023).

According to the National Institute of Health, “Reiki is a complementary health approach in which practitioners place their hands lightly on or just above a person, with the goal of directing energy to help facilitate the person’s own healing response” (NIH, 2018). Reiki practitioners describe the practice as working with universal, life force, energy (known as “chi,” “qi,” and “prana” in other cultures), by means of focusing the mind and forming an intention. It is generally accepted among practitioners that Reiki helps clear, balance, and fortify the bioenergetic field, removing energetic blocks that inhibit optimal flow and overall wellbeing.

The system of Reiki was developed in Japan in 1922 by Mikao Usui as a means to progress towards personal enlightenment. His system involved daily practices such as meditation, contemplation of five Reiki Principles, and offering hands-on Reiki to self and others. Teachers passed Reiki on to students via a course of study including the critical element of Reiju, or blessing, which is a ceremony intended to pass Reiki from teacher to student.

In the West, the practice of Reiki is sometimes seen more as a means of offering hands-on healing, and less as the spiritual path to enlightenment that Mikao Usui initially intended. This distinction may be attributed to Western cultural differences, the training one received, and/or one’s disposition towards service to others. Either way, Reiki is a safe and gentle practice that can be learned by most anyone over the course of a couple days (McManus, 2017). It is important to note that while Reiki is generally considered a spiritual practice, it is not affiliated with any religion, and is practiced by people of many faiths (McManus, 2017).

As the practice of Reiki has become more popular, so has interest in exploring its value as an intervention. Clinical trials evaluating the potential benefits of Reiki and their findings have been published in peer-reviewed journals ranging from those specific to complementary and energy healing modalities with no H Index score, all the way up to The Journal of the American College of Cardiology, with an H Index of 493 at the time of this writing (Friedman et al., 2010).

These trials have been conducted to evaluate Reiki’s impact on a wide range of parameters, and results have been significantly favorable for reducing anxiety (Curtiss et al., 2021; Hoge et al., 2023; McManus, 2017; Avcı and Gün, 2024; Baldwin et al., 2017; Başgöl et al., 2025; Bondi et al., 2021; Buyukbayram Genc and Citlik Saritas, 2024; Dyer et al., 2024; Fleisher et al., 2014; Gökdere Çinar et al., 2023; Graziano and Luigi, 2022; Oz Kahveci et al., 2025; Libretto et al., 2015; Ünal Aslan and Çetinkaya, 2024; Unal et al., 2024; Utli and Doğru, 2023; Vergo et al., 2018), fatigue (Buyukbayram Genc and Citlik Saritas, 2024; Dyer et al., 2024; Fleisher et al., 2014; Vergo et al., 2018; Bahçecioğlu Turan et al., 2024; Chen et al., 2023; Karaman and Tan, 2021; Koçoğlu and Zincir, 2021; Utli et al., 2023; Zins et al., 2019), depression (Fleisher et al., 2014; Graziano and Luigi, 2022; Libretto et al., 2015; Vergo et al., 2018; Zins et al., 2019; Charkhandeh et al., 2016); and improving quality of life (Astill Wright et al., 2019; Gökdere Çinar et al., 2023; Chen et al., 2023; Karaman and Tan, 2021; Koçoğlu and Zincir, 2021; Alarcão and Fonseca, 2016; Arıkan and Bahçecioğlu Turan, 2024; Thrane et al., 2022), sleep quality (Ünal Aslan and Çetinkaya, 2024; Bahçecioğlu Turan et al., 2024; Arıkan and Bahçecioğlu Turan, 2024; Costa et al., 2022), and vital signs (Ünal Aslan and Çetinkaya, 2024; Bahçecioğlu Turan et al., 2024; Arıkan and Bahçecioğlu Turan, 2024; Costa et al., 2022). Many of these studies were conducted to evaluate the use of Reiki in conjunction with medical procedures such as knee surgery or episiotomy (Friedman et al., 2010; Avcı and Gün, 2024; Baldwin et al., 2017; Buyukbayram Genc and Citlik Saritas, 2024; Utli and Doğru, 2023; Sagkal Midilli and Ciray Gunduzoglu, 2016; Notte et al., 2016; Topdemir and Saritas, 2021; Utli and Yağmur, 2022; Iacorossi et al., 2017; Aydemir et al., 2024), or in conjunction with cancer treatment (Dyer et al., 2024; Fleisher et al., 2014; Oz Kahveci et al., 2025; Chen et al., 2023; Karaman and Tan, 2021; Utli et al., 2023; Alarcão and Fonseca, 2016; Iacorossi et al., 2017; Özcan Yüce and Taşcı, 2021) or palliative care (Utli et al., 2023; Thrane et al., 2022; Thrane et al., 2021).

Several clinical trials have found Reiki to have a significant, favorable impact on stress (Bryant, 2022; Fritz et al., 2021; Connors, 2024; Curtiss et al., 2021; Astill Wright et al., 2019; Hoge et al., 2023; McManus, 2017; Fleisher et al., 2014; Oz Kahveci et al., 2025; Libretto et al., 2015; Unal et al., 2024; Utli and Doğru, 2023; Thrane et al., 2022; Özcan Yüce and Taşcı, 2021; Barut et al., 2024; Bukowski, 2015; Hailey et al., 2022; Mayra Del Carmen et al., 2023; Morimitsu et al., 2024; Vasudev and Shastri, 2016) and pain (McManus, 2017; Avcı and Gün, 2024; Baldwin et al., 2017; Bondi et al., 2021; Dyer et al., 2024; Fleisher et al., 2014; Gökdere Çinar et al., 2023; Graziano and Luigi, 2022; Oz Kahveci et al., 2025; Libretto et al., 2015; Vergo et al., 2018; Koçoğlu and Zincir, 2021; Utli et al., 2023; Zins et al., 2019; Thrane et al., 2022; Sagkal Midilli and Ciray Gunduzoglu, 2016; Notte et al., 2016; Topdemir and Saritas, 2021; Utli and Yağmur, 2022; Iacorossi et al., 2017; Aydemir et al., 2024; Jahantiqh et al., 2018; Unal et al., 2024). A study by Wyns A. et al. looked at patients with chronic pain and found a correlation between stress and increase in pain symptoms and stress-induced sensitivity to pain. “In fact, stress and pain are highly comorbid, and show significant overlap in both conceptual and biological processes” (Wyns et al., 2023; Abdallah and Geha, 2017). They found that individuals experiencing stressful circumstances were more likely to develop chronic pain, and patients with PTSD symptoms were more likely to report higher levels of pain (Generaal et al., 2016; Åkerblom et al., 2018). Conversely, individuals with chronic pain were more likely to develop conditions such as depression and anxiety (Yalcin and Barrot, 2014). This helped us understand why the high-stress communities we studied may have reported such high pain levels at the onset.

The International Association of Reiki Professionals developed a study, “America’s Best Hospitals,” which states “60% of the top hospitals had formal or informal Reiki programs in place. All hospitals using Reiki reported that Reiki is at least ‘somewhat beneficial’ for patients, and 67% of hospitals rated Reiki to be ‘highly beneficial’” (Dyer et al., 2024; IARP, 2014).

A paper developed for the United States military cites the following top U.S. hospitals as including a Reiki program and the year of program’s inception (Lanoy, 2015):

**Table:** 

Year program established	Hospital
1998	George Washington University Hospital
2001	Mayo Clinic
2002	Allegheny General Hospital
2002	Cleveland Clinic
2005	Yale-New Haven Hospital
2009	Brigham and Women’s Hospital (Harvard Medical School)

Other studies have found Reiki to be significantly beneficial to people in stressful life situations who are not themselves receiving allopathic healthcare, but rather undergoing challenging life circumstances (mothers with sick children, members of military branches, etc.) (Fritz et al., 2021; Başgöl et al., 2025; Libretto et al., 2015; Unal et al., 2024; Costa et al., 2022; Özcan Yüce and Taşcı, 2021; Hailey et al., 2022; Mayra Del Carmen et al., 2023; Morimitsu et al., 2024; Vasudev and Shastri, 2016).

The nature of this study is exploratory and does not intend to establish causality and clinical efficacy. The main purpose of this paper is to explore perceived stress and pain levels among high-stress communities in the Chicagoland area, before and after a ten-minute Reiki session, using self reported measures.

## 2 Context

This ongoing Reiki volunteer effort began in 2011 to support groups under excessive stress: first responders, first responders in training, homeless veterans, academic communities during stressful events, community violence intervention professionals and at-risk communities, individuals involved in the correctional system, and others. After 10 years and more than 5,000, ten-minute Reiki sessions, the group began to gather pre- and post-session data via de-identified surveys to better quantify the impact of the sessions.

Between September, 2022 and December, 2024, Reiki sessions were provided to 1,724 members of high-stress communities throughout the Chicago metropolitan area. The purpose of this paper is to analyze the data gathered during this window, which assessed self-reported levels of stress and pain before and after ten-minute Reiki sessions. While groups of participants were selected based on perceived stress levels, reductions in pain had been reported by participants for the duration of the program, so pre- and post-session pain levels were also recorded.

While other Reiki studies have been more rigorous in their methodology, very few have been of this scale. This study is also unusual in its use of shorter sessions taking place in non-clinical settings, factors which make it easily replicated by trained Reiki practitioners in the general population to support their community.

## 3 Methods and materials

### 3.1 Study design

**Figure 1:**
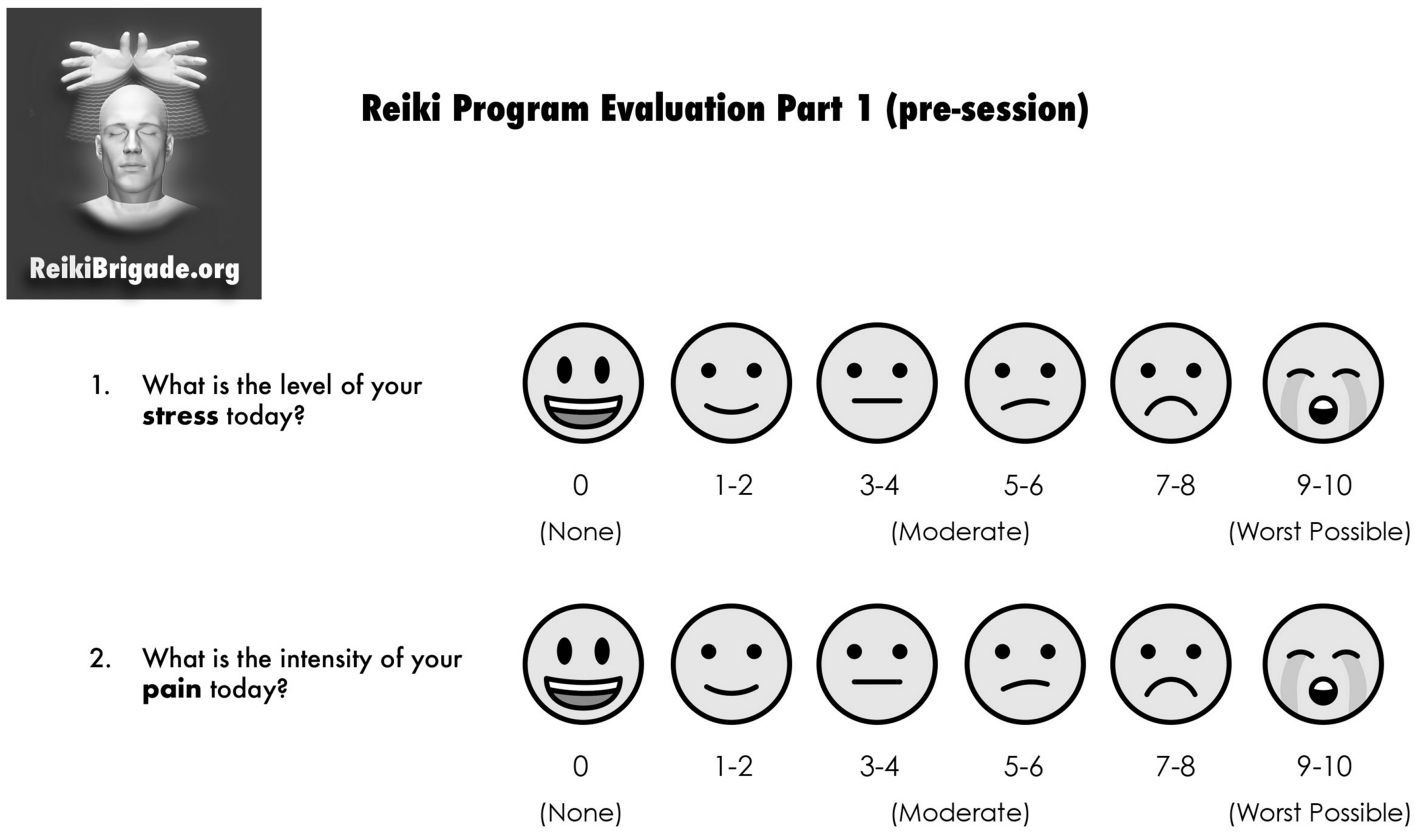
Pre-intervention survey. Emojis taken from OpenMoji – the open-source emoji and icon project. License: CC BY-SA 4.0.

**Figure 2:**
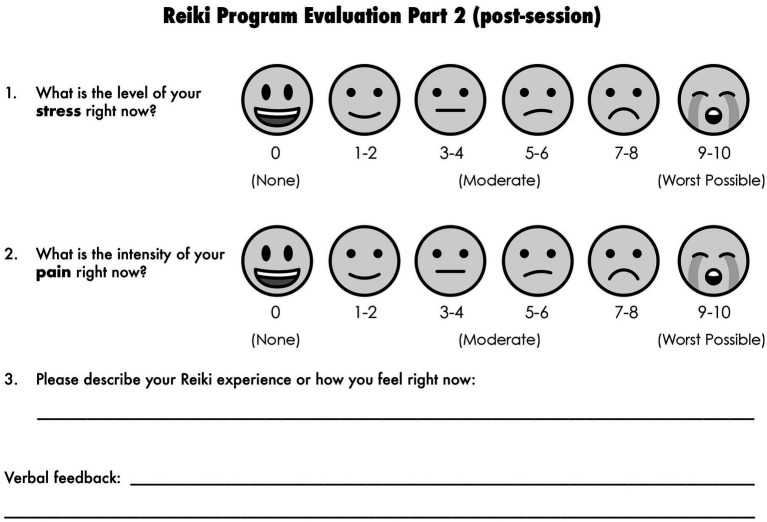
Post-intervention survey. Emojis taken from OpenMoji – the open-source emoji and icon project. License: CC BY-SA 4.0.

This qualitative, exploratory study was based on a pre-post observational intervention design used to evaluate the impact of ten-minute Reiki sessions on participants’ self-reported stress and pain levels. The study analyzed data collected from September 2022–December 2024 in Chicago. Written consents were taken from all the participants before the start of every session. Parent/Guardian consent was required for participants less than 18 years of age. Each participant received a ten-minute Reiki session. All participants received the intervention; there was no randomization or control group. Prior to each Reiki session, participants were asked to complete the pre-intervention survey (see Figure 1). After each session, participants were asked an open-ended question and then asked to complete the post-intervention survey (see Figure 2).

### 3.2 Study participants

Participant groups were selected based on belonging to a community experiencing higher-than-average stress due to their professions or life circumstances. Inclusion criteria of the study were any member of the community at that setting. Community members who were below 18 years of age without a guardian’s consent were excluded. Groups served were first responders, first responders-in-training, veterans, academic communities, Community Violence Intervention (CVI) & at-risk communities, and people in the correctional system. Beyond their affiliation with the community served, participants’ demographic information such as age, gender, socioeconomic status, or physical and mental health conditions were not gathered.

### 3.3 Settings

**Figure 3:**
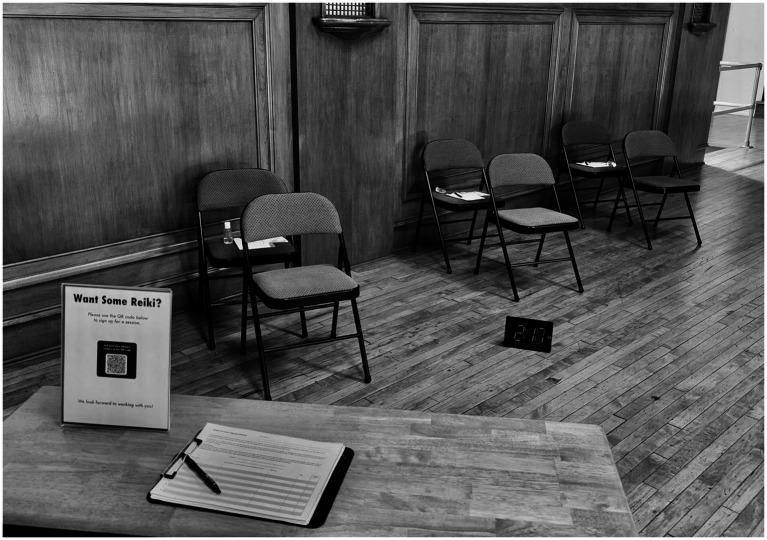
Reiki stations in a group setting.

Fifty-nine Reiki events were conducted in the Police Department, Fire Department, police and fire training academies, different college and university campuses, both indoor and outdoor wellness fairs, non-profit office spaces, and the Juvenile Detention Center in Chicago. Events were mostly three-hour, Reiki-specific events, and included a few, multi-faceted wellness fairs that lasted longer. The venues and atmospheres varied widely from a dedicated room with soft lighting and soothing music, to loud events open to the general public with big crowds, DJs, kids’ games, and food vendors. In each instance, a banner was set up outside the space to invite members of the community to participate and inform them of what was taking place. Each space was set up with a registration table and two regular chairs for each Reiki station, one for the practitioner and one for the recipient. All sessions were conducted in a group setting (see Figure 3).

### 3.4 Recruitment

Reiki was presented as a wellness benefit for the participant community and promoted via signage, and internal messaging when possible. Participants may have been new to Reiki or, in the case of an ongoing program within a venue, they may have been recurring participants.

### 3.5 Procedure

The practitioner gave a short explanation of Reiki and described how the session would proceed. Participants were given the option to receive Reiki via gentle touch or by hovering in the energy field without direct contact. All but a very few participants granted permission for physical touch. Participants are asked to indicate on the pre-intervention component of the survey how they are feeling, in that moment, in terms of stress and pain by marking the scales. Following completion of the pre-intervention component of the survey, participants were invited to close their eyes, take a couple deep breaths, and relax into the chair. All sessions involving physical contact began with the practitioners’ hands on the participants’ shoulders and ended by offering Reiki to their feet.

**Figure 4:**
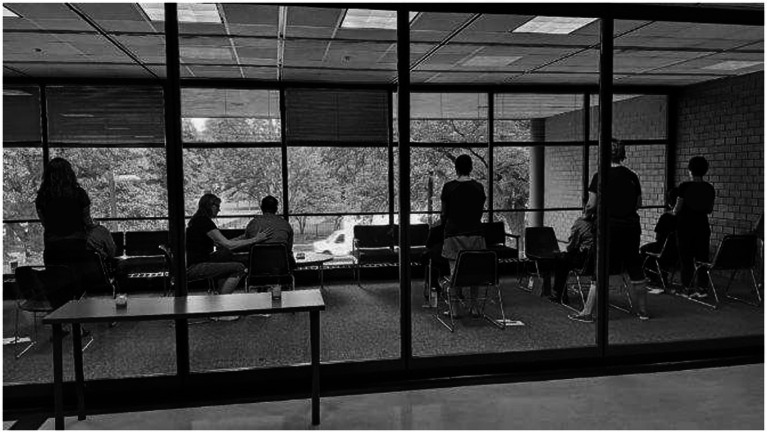
Reiki sessions in process.

Sessions involving only hovering proceeded similarly, with hands a few inches above the body, rather than in direct contact. Other hand positions generally included head, upper back, and knees (see Figure 4). Additional hand positions were offered based on initial responses to the survey questions, for example, low back pain would be addressed by spending a few minutes at the participant’s low back as part of the progression. For sessions where stress was the primary complaint, more time was spent on the participants head, shoulders and over the heart, and less time at the lower extremities.

Post-intervention, participants were asked open-ended questions such as, “How do you feel?” or “How did that go?” and their feedback was recorded. They were then offered the post-intervention component of the survey and asked to record their stress and pain levels at that moment.

### 3.6 Data collection/instruments

Both quantitative and qualitative data were collected using subjective measurement tools. Quantitative measures were determined using a 1–10 scale with facial expressions as a visual aide (see Figures 1, 2). This scale was selected for ease of understanding and to help participants navigate the form quickly. Qualitative data was collected post-intervention by asking participants open-ended questions such as, “How do you feel?” or “How did that go?” and their feedback was recorded. There was also space on the form for participants to write their own comments following the prompt, “Describe your Reiki experience or how you feel right now.” No structured interview guide was followed, and no differentiation was made between verbal comments and written comments. All data was de-identified to ensure confidentiality.

### 3.7 Practitioners

Thirty-one volunteer practitioners participated in the program during the data collection window. They included females (29) and males (2), ranging in age from 37 to 79. Applicants’ experience with Reiki ranged from those recently completing a Level 1 class, to Reiki Masters who had practiced for more than a decade either professionally or informally. All applicants also received specific training to cover the volunteer program’s policies and procedures by the program director.

### 3.8 Data analysis

For quantitative data, descriptive statistics including the mean and percentage of change in stress and pain levels were calculated in Microsoft Excel. *p*-value was calculated in R programming to test the significance of the study. Due to limited variables, other statistical analysis was not done. Qualitative data from open ended questions were generated using a word frequency analyser (Word Frequency, 2025) which tabulated the frequency of word use in feedback comments. No other software or coding tools were used to analyse the themes. Responses from the participants were categorized into common themes and individual groups’ compelling comments. All the data were de-identified during analysis.

### 3.9 Ethics approval statement

Written informed consent was obtained from all the participants prior to the start of the Reiki session. Although all the data were de-identified, ethical approval was obtained from an Northeastern Illinois University Institutional Review Board (Protocol #335) to publish the findings.

## 4 Results

A total of 1,724 people who received a ten-minute Reiki session were included in the study. Survey responses were analyzed separately for both stress and pain variables (Table 1).

**Table 1:** Total number of participants in each variable Stress and Pain.

Variable	Total complete surveys	Total incomplete surveys	Total # of participants
Stress	1,596 (92.6%)	128 (7.4%)	1,724
Pain	1,544 (89.6%)	180 (10.4%)	

This table represents number and percentage of study participants who had completed or not completed the survey for both pain and stress variables, and the total number of participants.

For stress: Of the total participants 1,596 (92.6%) completed the survey and 128 (7.4%) provided incomplete responses.

For pain: Of the total participants 1,544 (89.6%) completed the survey and 180 (10.4%) provided incomplete responses.

Only participants who completed both pre- and post-intervention surveys were included in analysing the data. Incomplete responses were excluded from the analysis.

The completion rate was found to be higher for the stress variable when compared to pain. Cumulative results of pre- and post-interventions of stress and pain variables are shown in Table 2. Both end results were found to be statistically significant with *p* value of < 0.01.

**Table 2:** Cumulative results of total number of participants.

Variable	Pre-intervention survey results	Post-intervention survey results	Change (%)	*p*-value
Stress	4.31	1.22	3.13 (72.62%)	< 0.01
Pain	3.11	1.10	1.97 (63.34%)	< 0.01

This table indicates the cumulative survey results of stress and pain levels before and after the 10-min Reiki intervention among the study participants. The values represent the average score, change in percentage, and the *p*-value for statistical significance of the intervention.

Reiki sessions were conducted in 59 different settings. Settings were divided into six groups to analyze the results (Table 3).

**Table 3:** Total number of participants in each group.

Group	Stress		Pain		Total number of participants
	No. of complete surveys	No. of incomplete surveys	No. of complete surveys	No. of incomplete surveys	
First Responders	474	26	463	37	500
First Responders-in-Training	282	21	267	36	303
Veterans	90	6	88	8	96
Academic Communities	350	25	328	47	375
CVI & At-Risk Communities	314	43	310	47	357
Corrections	86	7	88	5	93

This table represents the distribution of participants into each group for both stress and pain variables showing the number of complete and incomplete surveys with the final column indicating the total number of participants in each group.

Group 1: First Responders—Participants were primarily police officers, firefighters, and departmental staff, along with a few family members at a Family Wellness Day for First Responders.

Group 2: First Responders in Training—Participants were primarily police and fire department recruits in training at their respective academies, along with some instructors and staff.

Group 3: Veterans—Participants were attendees of Stand Down service fairs for homeless veterans and included a small number of other service providers (many of whom are also veterans), and active military who supported event operations.

Group 4: Academic Communities—Participants included college and university students during exams or recruitment interviews; Chicago Public School teachers and counselors; and a few university faculty members and other vendors.

Group 5: CVI & at-risk communities—Participants were all in under-resourced neighborhoods in Chicago and included staff of CVI organizations, the majority of whom come from the communities they serve and have significant lived experience with gun violence; at-risk youth; foster parents and children; and attendees of community wellness/gun violence intervention events.

Group 6: Corrections—Participants were individuals recently released from prison and staff members of a juvenile detention center.

The qualitative component is included in this paper to provide support for the quantitative results and to bring to light patterns of perceived impact of the Reiki sessions. Results of quantitative and qualitative feedback of individual groups are shown in Table 4. All the groups’ results in Table 4 were found to be statistically significant. The most common words identified through a word frequency analyser were 1. Relaxed (724); 2. Grateful (258); 3. Reduced Pain (110); 4. Sleepy (80); and 5. Amazing (70) (see Table 4). However as this analysis is based on word frequency rather than systematic coding, the qualitative response analysis is descriptive rather than in-depth. More compelling comments such as profound surprise at the positive outcomes, reference to religious experiences, and something being tangibly removed from their bodies were randomly picked from each group and are listed in Table 5.

**Table 4:** Occurrence of word frequency in qualitative feedback.

Forms of relax (724)	Forms of calm (258)	Forms of thank you (87)	Forms of sleep (80)
Relaxed (451)	Calm (163)	Thank (71)	Asleep (42)
Relaxing (246)	Calming (73)	Thanks (8)	Sleep (18)
Relax (27)	Calmer (19)	Thankful (6)	Sleepy (18)
	Calmness (3)	Thankyou (2)	Sleeping (2)

Pain (112)	Forms of Light (110)	Amazing (70)	Awesome (36)
Light (55)			
Lighter (55)			

This table represents common words used in qualitative responses, and their frequency tabulated using a word frequency analyser.

**Table 5:** Results of individual groups.

Group	Variable	Pre-intervention survey results	Post-intervention survey results	Change (%)	*p*-value
First responders	Stress	4.25	1.09	3.14 (73.88%)	< 0.01
	Pain	3.05	1.09	1.96 (64.26%)	< 0.01
	Qualitative feedback	“I felt like I was communing with God.” “It was a total reset—stress level zero!” “So relaxed I cannot even write.” “I love what you all have done for me today—no stress!” “Do people feel like this, or am I crazy?”			
First responders-in-training	Stress	4.21	1.06	3.15 (74.82%)	< 0.01
	Pain	2.80	0.94	1.86 (66.43%)	< 0.01
	Qualitative feedback	“I feel like my heart rate has slowed down.” “My head was spinning with thoughts, and that just went away.” “This lifted some weight off my shoulders.” “Stuff coming out of my head.” “Could feel the pain move out.”			
Veterans	Stress	4.51	1.75	2.76 (61.20%)	< 0.01
	Pain	4.36	2.17	2.19 (50.23%)	< 0.01
	Qualitative feedback	“It’s really awesome what you do. My knee pain is gone now. Thank you!” “Felt like my heart opened.” “I feel very settled. I have PTSD, so when you started my mind was immediately off and running, but I did not get caught up in it. I stayed at ease… and just kind of watched it, like I was watching a movie.” “Felt the pain going out of my body.” “I can feel a huge difference.”			
Academic communities	Stress	4.73	1.51	3.22 (68.08%)	< 0.01
	Pain	2.83	1.11	1.72 (60.78%)	< 0.01
	Qualitative feedback	“Something was pulled out of my chest.” “You have a superpower.” “This cannot possibly be real—but it is!” “I was skeptical, but wow! So glad I tried it!” “It’s a miracle.”			
CVI/at-risk communities	Stress	4.22	1.06	3.16 (74.88%)	< 0.01
	Pain	2.80	0.94	2.25 (80.36%)	< 0.01
	Qualitative feedback	“I do not know if I’m tripping or what, but it felt like you pulled something out of me.” “Amazing—even my back pain is gone.” “That was fantastic! I should get my blood pressure taken again now.” “I felt like I was talking to and touching God.” “Transcendental.”			
Corrections	Stress	4.08	1.17	2.91 (71.32%)	< 0.01
	Pain	2.46	1.21	2.07 (84.15%)	< 0.01
	Qualitative feedback	“Heart rate went down.” (per personal device) “I feel great. Need to try this at home. Pain gone completely.” “I feel like I released something.” “My bad energy just flew away.” “I felt stress flow right out of my body.”			

This table summarizes the results of both quantitative and qualitative data in each subgroup. Values indicate the average before and after results, percentage change, and the *p*-value.

**Figure 5:**
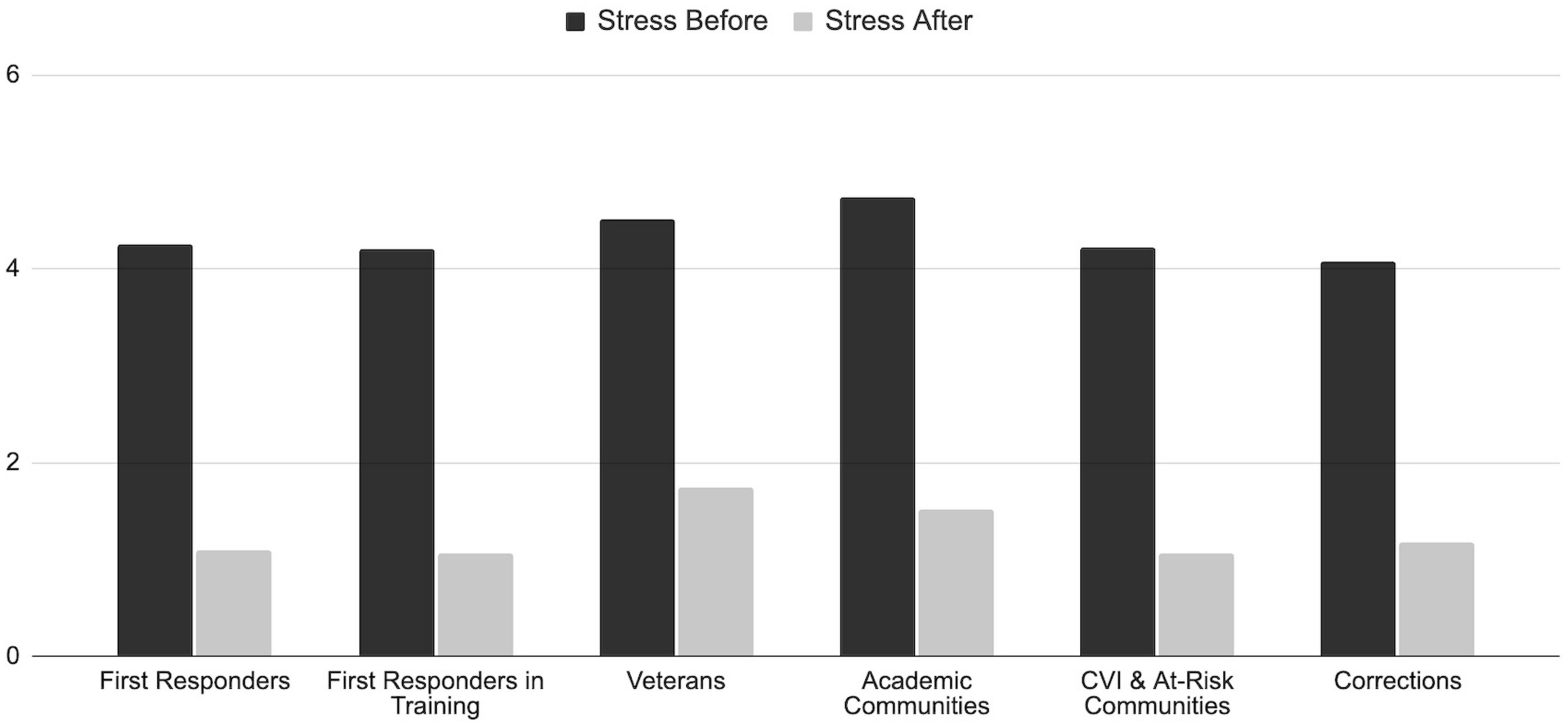
Change in reported stress level among the participants in each group before and after the 10-min Reiki intervention.

The change in level of stress before and after the Reiki session in each group are shown in Figure 5.

**Figure 6:**
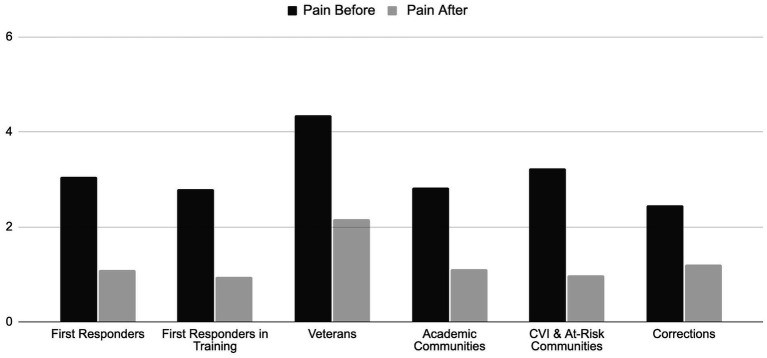
Change in reported pain level among the participants in each group before and after the 10-min Reiki intervention.

The change in level of pain before and after the Reiki session in each group are shown in Figure 6.

A comparison of survey results from events in settings with disparate ambience are shown in Table 6.

**Table 6:** Comparison of results from events in settings with disparate ambience.

Setting	Total number of participants	Variable	Pre-intervention survey results	Post-intervention survey results	Change (%)
Most relaxing ambience: dedicated room, soft lighting, soft music, aromatherapy	270	Stress	4.89	1.30	3.59 (73.42%)
	260	Pain	3.48	1.24	2.24 (64.37%)
	Qualitative feedback	“That was something. Wow. I felt a deep decompression and I am so relaxed.” “She took all my stress, anxiety and restlessness away. I was able to center my mind.” “I have an injured nerve and when you put your hand there I felt it pulsating. I felt like this calm come over me. I felt tingling all over my body. It was great.” “My mind and body began to ‘sync.’ I began to feel calm.” “I feel so relaxed. I definitely felt something. Your hands were not moving!? How soon can you guys come back?”			
Most disruptive ambience: outdoors in a festival-like environment, loud music, DJ	75	Stress	4.25	0.955	3.29 (77.41%)
	76	Pain	3.23	0.885	2.34 (72.45%)
	Qualitative feedback	“Amazing! Indescribable no words to describe it! Even though there was a lot of noise, my body just relaxed. Just amazing!” “Like a meditation—the bells of the kids’ bikes were like music. Really relaxing.” “I felt lighter more in time with the world.” “It was a moment of peace—just me & the universe.” “I forgot where I was. I really zoned out.”			
CPD shooting range: gun shots next door throughout the event	6	Stress	4.17	0.67	3.50 (83.93%)
	6	Pain	2.34	0.91	1.43 (61.11%)
	Qualitative feedback	“I feel wonderful! I have a sensation of calmness. All my pain is gone! I am so happy I came.” “It is like if my body was really settled. I have no severe stress. I feel really relaxed.” “I think I fell asleep. I think I was dreaming? I’m so relaxed.” “Oh I fell asleep! I feel so relaxed! My pain in my shoulder is gone. I could feel the energy moving out of my hands and my knee. This is so cool!” “I could feel my mind let go. I kind of drifted off. It’s been a really busy day. Feel so relaxed.”			
CPD high skepticism: relaxing ambience, but highly skeptical population	5	Stress	2.90	1.30	1.60 (55.17%)
	5	Pain	3.30	0.90	2.40 (72.7%)
	Qualitative feedback	“Relaxed.” “Refreshed.” “Calm.” “Very good.” “Can I come again?”			

This table compares settings with the most relaxing and disruptive ambience that might have impacted the findings after each Reiki intervention.

## 5 Discussion

This study describes patterns in participants’ self-reported perception following a brief ten-minute Reiki session among highly stressed individuals. Unlike more rigorous clinical trials, this exploratory, perception-based study limits the interpretability of the findings. However, its large scale and promising results can help generate hypotheses, and encourage and inform future research.

The findings suggest that even brief Reiki sessions may result in considerable reductions in stress and pain levels. Findings from the quantitative data showed significant reduction in self-reported stress (72.62%) and pain (63.34%) levels after a single session with a *p*-value of <0.01 across all groups. Qualitative feedback was overwhelmingly positive, supported the quantitative findings, and provided a more robust and comprehensive explanation of participants’ experiences.

The findings from this study are consistent with more methodologically rigorous, prior research in demonstrating the effectiveness of Reiki in reducing stress (Bryant, 2022; Fritz et al., 2021; Connors, 2024; Curtiss et al., 2021; Astill Wright et al., 2019; Hoge et al., 2023; McManus, 2017; Fleisher et al., 2014; Oz Kahveci et al., 2025; Libretto et al., 2015; Unal et al., 2024; Utli and Doğru, 2023; Thrane et al., 2022; Özcan Yüce and Taşcı, 2021; Barut et al., 2024; Bukowski, 2015; Hailey et al., 2022; Mayra Del Carmen et al., 2023; Morimitsu et al., 2024; Vasudev and Shastri, 2016) and pain (McManus, 2017; Avcı and Gün, 2024; Baldwin et al., 2017; Bondi et al., 2021; Dyer et al., 2024; Fleisher et al., 2014; Gökdere Çinar et al., 2023; Graziano and Luigi, 2022; Oz Kahveci et al., 2025; Libretto et al., 2015; Vergo et al., 2018; Koçoğlu and Zincir, 2021; Utli et al., 2023; Zins et al., 2019; Thrane et al., 2022; Sagkal Midilli and Ciray Gunduzoglu, 2016; Notte et al., 2016; Topdemir and Saritas, 2021; Utli and Yağmur, 2022; Iacorossi et al., 2017; Aydemir et al., 2024; Jahantiqh et al., 2018; Unal et al., 2024) levels. Unlike the majority of these studies, which evaluated the impact of longer Reiki sessions, this study utilized shorter sessions.

Additionally, this study includes a large number of participants from a diverse range of high-stress communities. A 2019 study by N. Dyer et al. conducted 1,411 Reiki sessions to evaluate the effectiveness of a single session of Reiki on physical and psychological health. They found multiple parameters related to overall health improved post-intervention. This is one of very few large-scale, published studies, and it was based on 45–90 min sessions, whereas our study evaluates the impact of ten-minute Reiki sessions (Dyer et al., 2019).

Almost all first-time participants presented with initial skepticism. Participation was often driven by the snowball effect, with early adopters recruiting their reluctant colleagues to participate based on their own, positive experiences.

Verbal feedback recorded by the practitioner was almost always more descriptive and compelling than written feedback provided by the participants. Based on this observation, it is strongly suggested that future studies take the extra step to gather verbal qualitative feedback in addition to any written feedback. Recurring themes in both forms of qualitative feedback include relaxation, calm, reduced pain, feeling lighter, awe/amazement, gratitude, and sleepiness. All these themes were indicated by a word frequency analyser. However, we acknowledge that absence of a structured thematic coding system limits the depth and rigor of the qualitative analysis. We do recommend future studies should consider structured interviews and formal thematic analysis in order to improve reliability and richness in qualitative findings.

### 5.1 Impact of setting

In comparing outcomes for settings with ambient disparities, we found the results were very similar, and that other factors may have had more impact on session outcomes. Two sets of events stand out as consistently having the most relaxing ambiance and the most disruptive ambiance. In this section we will compare the outcomes for these sets of events, as well as two outlier events in terms of setting and outcomes.

During seven events at Chicago Police Headquarters, sessions took place in a very relaxing setting: a dedicated room with dimmed lights, soft music, and aromatherapy. Participants (*n* = 270) reported an average 3.58 (73.42%) reduction in stress, and participants (*n* = 260) reported an average 2.24 (64.37%) reduction in pain.

During four events at an expo promoting safety in the streets (non-violence), sessions took place in a highly disruptive setting: outside in a festival-like atmosphere with a DJ, loud dance music, and other distractions. Participants (*n* = 75) reported an average 3.29 (77.41%) reduction in stress, and participants (*n* = 75) reported an average 2.34 (72.45%) reduction in pain.

During an event at an outlier setting inside a police district in a conference room adjacent to the shooting range sessions were given while gunshots continuously rang out next door. Five participants reported an average 3.50 (83.93%) reduction in stress and 1.43 (61.11%) reduction in pain. This was arguably the most disruptive setting of all and yet resulted in the highest percent reduction in reported stress.

The lowest pre- to post-intervention change in reported stress took place at a venue we visited only once. While the setting was ambient with a dedicated room, soft lighting, and music, the five participants reported an average 1.60 (55.17%) reduction in stress. Qualitative feedback was much less descriptive and enthusiastic than comments at other events. Skepticism and negative peer pressure from the community of potential participants seemed to be a significant factor in outcomes at this event.

Based on these findings, it appears that setting and ambiance had little bearing on the outcomes of the Reiki sessions. These findings correspond with those in a previous study designed to measure the impact of Reiki sessions in a loud mall with artificial lighting and noise, in other words, without any ambient support (Graziano and Luigi, 2022). Conversely, the attitudes and peer pressure of the group being offered the intervention seemed to have a more marked impact. This suggests a broader conversation, and perhaps a future study, about the availability and impact of Reiki in the face of public skepticism.

### 5.2 Additional physiological benefits of Reiki sessions

In a study by Díaz-Roderíguez et al., later cited by Daviu N. et al., it was found that, “…a single session of Reiki increased heart rate variability and body temperature but not salivary cortisol levels, indicating that Reiki shifts the autonomic balance toward parasympathetic dominance” (Daviu et al., 2019; Díaz-Rodríguez et al., 2011). The parasympathetic nervous system and sympathetic nervous system work together to regulate the autonomic nervous system which controls involuntary bodily functions. The sympathetic nervous system is activated, the individual is said to be in “fight or flight,” as the body responds to danger. When the parasympathetic nervous system is activated, the individual is said to be in “rest and digest,” as the body returns to a state of relaxation (Parasympathetic Nervous System, 2025).

While both states are important, the complementary dynamic between the two allows the body to maintain healthy function while adapting to changing circumstances when necessary. Fluidity and balance in this system are key for optimal health. People who are experiencing high levels of stress on an ongoing basis may experience an imbalance favoring sympathetic activation, resulting in health issues related to the dysregulation of basic bodily functions including respiration, digestion, and heart rate. The ability to shift the autonomic nervous systems towards parasympathetic, as a single Reiki session has been shown to do, is believed to benefit overall wellbeing at fundamental physiological levels (Parasympathetic Nervous System, 2025).

Reiki can be learned quickly and easily by most anyone and used as both a form of self-care and an intervention for others. While the sessions in this program were conducted by volunteers, Reiki is often a paid service offered by professional practitioners much in the same way massage is provided. Unlike massage, Reiki has no governing body in the U.S. While more and more information is available to the general public via published, clinical trials, news outlets, books, podcasts, conferences, and social media, training is done in a variety of ways and the duration, content, and quality of classes is not standardized or regulated in any way. People seeking Reiki services or training are encouraged to do research to find competent practitioners and instructors.

On-site Reiki programs are open to everyone in a community, reduce barriers to care in that they are accessible, extremely cost-effective, and help eliminate stigma associated with seeking out mental health treatment.

## 6 Limitations

This study has several significant limitations that should be considered. The data gathered during this program was not initially intended for a research study, but rather to report the participants’ feedback from the sessions back to the communities served.

As the study is a qualitative, exploratory, pre-post design, all the findings are solely based on self-reported perceptions. This study also lacks a control group or randomization which increases the risk of potential bias.

There was no standardized scale used to assess the stress and pain levels. The use of subjective, 1–10 scales with visuals used for both stress and pain levels, while practical and easy to administer, may not capture the full clinical relevance of these variables.

The absence of demographics data such as age, gender, socioeconomic status, and disability status restricts the generalizability of the results. Additionally, limited variables (e.g., stress and pain) further limits the ability to conduct more in-depth statistical analysis.

The qualitative component, which consisted of very brief, open-ended feedback without any structural interview guide or systematic coding, limited the rigor of the qualitative analysis. We also recognize that having the practitioners administer the surveys leaves room for participant bias. Ideally, surveys would be administered independently from practitioners.

It should be noted that social interactions inherent to the process, participant expectations, and placebo effects could have influenced the outcomes as much as the Reiki intervention itself. This is an aspect that can be addressed in future studies using control groups, randomization, and where possible, blinding study methods.

Going forward, we will gather demographic data such as age and gender of participants and determine how many of the participants are first-time Reiki recipients. We will also consider capturing data such as physical and mental health conditions, as well as considering how we can structure a future study utilizing a control group while retaining ethical Reiki practices.

## 7 Conclusion

This exploratory study sheds light on how participants in high-stress communities perceive positive changes in their stress and pain levels following a brief Reiki session. Due to the study’s limited design, the results reflect subjective experiences and, more importantly, should not be interpreted as objective clinical effects. However, they provide valuable exploratory insights into how participants perceive brief Reiki sessions—particularly in high-stress community settings, and lay the groundwork for future, controlled studies that can more rigorously evaluate Reiki interventions, using standardized measures and objective indicators.

This study demonstrates that participants chosen for belonging to high-stress populations reported statistically significant improvements in both stress (72.62%) and pain (63.34%) following a single, ten-minute Reiki session. Qualitative feedback supported quantitative data with regard to participants feeling very relaxed and experiencing less pain. Themes among more compelling qualitative responses include profound surprise at the positive outcomes and reports of tangible experiences beyond the tactile sensations of practitioner contact, including feeling lighter.

These results suggest potential benefits, however because of the study’s limitations, they should be interpreted with caution.

We encourage replication of this study, specifically in high-stress communities in various settings, using standardized scales, structured interviews, and better demographic data collection. Further research is needed to determine longer-term effects of repeated Reiki sessions. This research can be conducted by Reiki-trained individuals throughout the general population in both clinical and non-clinical settings. This underscores Reiki’s potential as a safe, non-invasive, cost effective, and highly accessible—yet effective—intervention.

It is strongly recommended that verbal responses be elicited and recorded following sessions to continue to document recipients’ experiences during and following Reiki sessions.

## Data Availability

The raw data supporting the conclusions of this article will be made available by the authors, without undue reservation.
